# Invariant Natural Killer T and Mucosal-Associated Invariant T Cells in Asthmatic Patients

**DOI:** 10.3389/fimmu.2018.01766

**Published:** 2018-07-30

**Authors:** Guillaume Lezmi, Maria Leite-de-Moraes

**Affiliations:** ^1^AP-HP, Hôpital Necker-Enfants Malades, Service de Pneumologie et d’Allergologie Pédiatriques, Paris, France; ^2^Université Paris Descartes, Paris, France; ^3^Laboratory of Immunoregulation and Immunopathology, INEM (Institut Necker-Enfants Malades), CNRS UMR8253 and INSERM UMR1151, Paris, France

**Keywords:** asthma, NKT cells, mucosal-associated invariant T cells, children, patients, CD1d, MR1, Th2 cells

## Abstract

Recent studies have highlighted the heterogeneity of asthma. Distinct patient phenotypes (symptoms, age at onset, atopy, and lung function) may result from different pathogenic mechanisms, including airway inflammation, remodeling, and immune and metabolic pathways in a specific microbial environment. These features, which define the asthma endotype, may have significant consequences for the development and progression of the disease. Asthma is generally associated with Th2 cells, which produce a panel of cytokines (IL-4, IL-5, IL-13) that act in synergy to drive lung inflammatory responses, mucus secretion, IgE production, and fibrosis, causing the characteristic symptoms of asthma. In addition to conventional CD4^+^ T lymphocytes, other T-cell types can produce Th2 or Th17 cytokines rapidly. Promising candidate cells for studies of the mechanisms underlying the pathophysiology of asthma are unconventional T lymphocytes, such as invariant natural killer T (iNKT) and mucosal-associated invariant T (MAIT) cells. This review provides an overview of our current understanding of the impact of iNKT and MAIT cells on asthmatic inflammation, focusing particularly on pediatric asthma.

## Introduction

Asthma is now considered to encompass different conditions characterized by common symptoms (wheeze, cough, shortness of breath, and chest tightness), variable degrees of airflow limitation, and different pattern of inflammation. Most patients with asthma have an eosinophilic infiltration of the airways, associated with increased production of type 2 cytokines including IL-4, IL-5, IL-13 secreted by Th2 cells, together with allergic comorbidities (1). However, around 50% of adults with asthma do not fall into this description (2). Asthmatic patients with a neutrophil-high signature were described in both adults and children (3–5). This neutrophilic-predominant endotype is less well understood than the Th2 endotype and may be related to the activation of the IL-17 pathway (1, 6). Intriguingly, despite eosinophilic airway inflammation is a key feature of severe asthma in schoolchildren, there is no clear evidence for a Th2 type cytokine signature in bronchial mucosa or bronchoalveolar lavages in that population (7, 8). Alternative mechanisms may, therefore, be involved in the pathogenesis of asthma in this group. Recent studies have suggested the potential role of unconventional T cells, such as invariant natural killer T (iNKT) and mucosal-associated invariant T (MAIT) cells in asthma pathogenesis. These T lymphocytes usually reside in the tissues, including those of the airways and can respond rapidly to stimuli by producing Th2 and Th17 cytokines. Here, we review the field of asthma immunity, focusing on the role of iNKT and MAIT cells in asthmatic patients.

## iNKT Cells

The major characteristic of iNKT lymphocytes is their expression of T cell receptors of limited diversity recognizing lipid antigens presented by the non-polymorphic MHC-like molecules, CD1d (9, 10). iNKT cells express an invariant TCRα chain, Vα14-Jα18 (or TRAV11 TRAJ18) in mouse, and Vα24-Jα18 (or TRAV10 TRAJ18) in humans, together with a limited set of TCRβ chains (10, 11). The invariant TCRα chains of mice and human are very similar, enabling the iNKT cells to recognize the same glycolipids in both species. A classic example is provided by α-galactosylceramide (α-GalCer), an antigen capable of stimulating both mouse and human iNKT cells (12, 13). α-GalCer and its analog, PBS57, are currently used as antigens for the production of a CD1d-tetramer complex capable of specifically identifying iNKT cells (14). iNKT lymphocytes respond rapidly to specific lipid antigens, in a TCR-dependent manner, but they also respond to pro-inflammatory cytokines (12, 15, 16). Indeed, IL-12 and IL-18 induce the production of IFNγ, whereas IL-1β and IL-23 will promote the secretion of IL-17A (or IL-17) and IL-22 (17, 18). Further, iNKT cells have been shown to express IL-25 (or IL-17E), thymic stromal lymphopoietin, and IL-33 receptors that will favor their secretion of IL-4, IL-13, and IFNγ (19–22). Human iNKT cells require TGFβ for the production of IL-17 and IL-22 (23). TCR-dependent and TCR-independent pathways can act in synergy to stimulate iNKT cells more strongly (15, 24).

**Figure 1:**
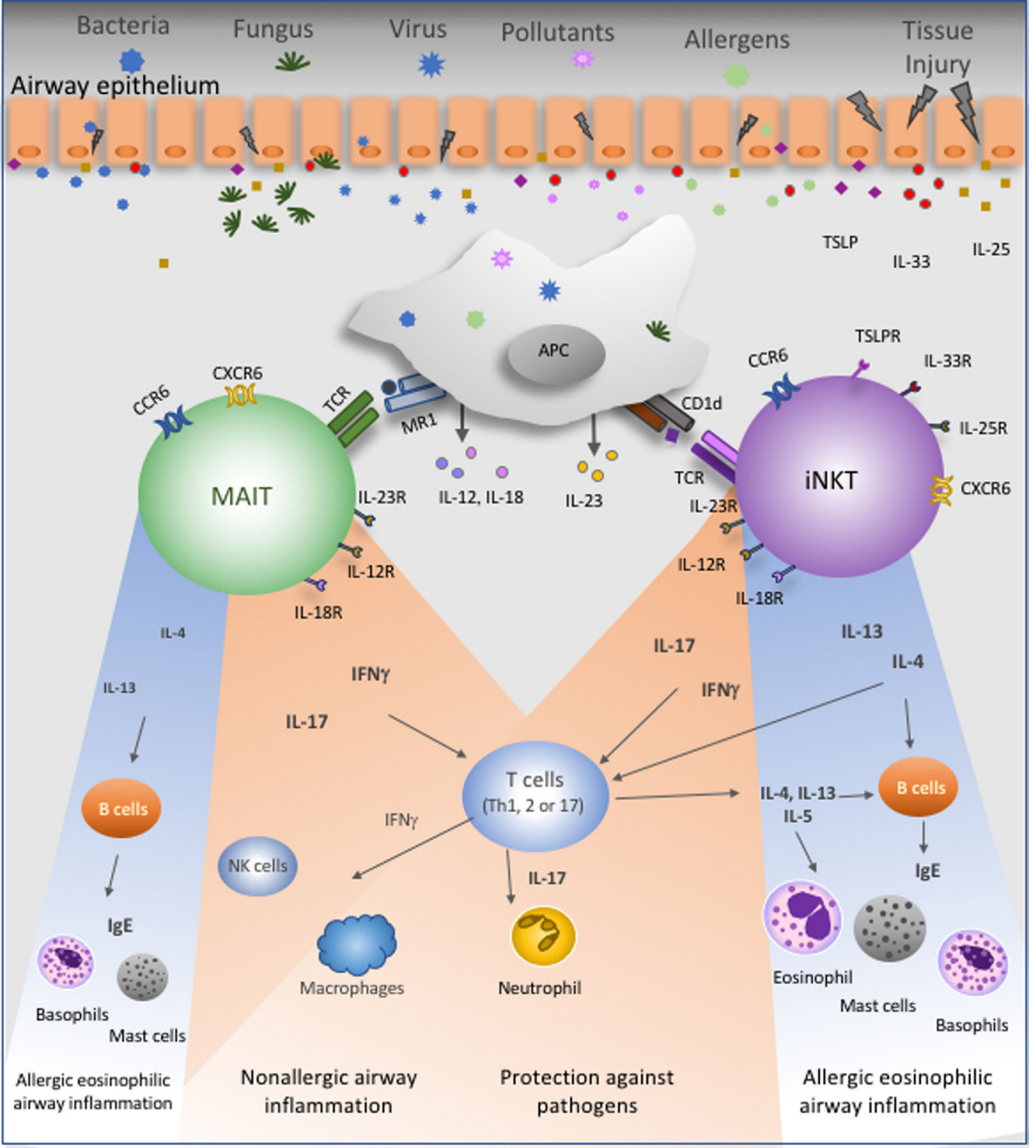
Proposed roles of mucosal-associated invariant T (MAIT) and invariant natural killer T (iNKT) cells in the lung. These unconventional T lymphocytes are present in the lung at steady state and express several chemokine- and interleukin-receptors. Bacteria, fungus, virus, pollutants, and airway allergens will directly or indirectly stimulate MAIT and iNKT cells. Cytokines produced by epithelial cells, namely, IL-25, IL-33, and thymic stromal lymphopoietin, could activate these cells. Antigen-presenting cells (APC) present antigens to MAIT and iNKT cells in the context of MR1 and CD1d molecules, respectively. Activated APC produce IL-12, IL-18, and IL-23 that will stimulate MAIT and/or iNKT cells. Following TCR-dependent or TCR-independent activation, MAIT and iNKT cells secrete IFNγ, IL-17, IL-4, or IL-13. IFNγ contributes to lung protection and promotes potential protective Th1 responses against asthma. IL-17, in turn, could have a dual effect since it is known that this cytokine promotes neutrophils recruitment and activation to protect lung from injury, but IL-17 can also enhance neutrophilic asthma severity. Finally, IL-4 and IL-13 will favor Th2 immune responses and then amplify allergic eosinophilic airway inflammation.

The thymic differentiation of iNKT cells is tightly controlled. At least three major iNKT cell subsets mature in the thymus, the iNKT1 (IFNγ and IL-4 producers), the iNKT2 (IL-4 and IL-13 producers), and the iNKT17 (IL-17 and IL-22 producers) (25–28). Maturation in the thymus is regulated by the Slam-Associated Protein (29), the transcription factors PLZF, Egr2, ThPOK, Runx1, and RORγt, the microRNA Let-7, and the cytokine IL-7 (30–38). iNKT cells undergo several maturation steps [see Ref. (10, 35) for more information], before migrating to peripheral organs as CD4^+^CD8^−^ and double-negative (CD4^−^CD8^−^) cells. In humans, there is also a CD4^−^CD8^+^ subset (39). iNKT cells are mostly resident in tissues, where they can “patrol” to identify threats to the body. For instance, these cells have been shown to perform an intravascular immune surveillance function in the liver, spleen, and lung (40–43). The primary function of iNKT cells is to protect the host from infections (24, 44, 45). However, in some conditions, iNKT cell activation favors tissue injury, including lung (Figure 1), as discussed below.

### iNKT Cells and Murine Asthma Models

Mouse models are widely used to help clarify the role of iNKT cells in asthma. Studies initially focused on allergic asthma, with ovalbumin (OVA) as the allergen, associated with aluminum hydroxide as adjuvant, for the systemic immunization followed by intranasal (i.n.) OVA challenge. First analysis showed no major difference in the severity of allergic airway inflammation in β_2_microglobulin (β_2_m)^−/−^ and CD1d^−/−^ mice, which lack iNKT cells (46–48). However, other studies reported that iNKT cell-deficient (Jα18^−/−^ and CD1d^−/−^) mice had attenuated asthma symptoms including airway hyperresponsiveness (49), airway eosinophilia, Th2 inflammation, and OVA-specific anti-IgE production (50, 51). The adoptive transfer of IL-4- and IL-13-producing iNKT cells restored the asthma severity, demonstrating that iNKT cells favored allergic asthma symptoms through the production of these cytokines (50, 51). iNKT cells did not recognize OVA as an antigen, but their ability to promote lung inflammation was reduced by the treatment of mice with anti-CD1d antibodies, indicating that endogenous lipidic antigens stimulated the iNKT cells (50). More recently, another study comparing distinct iNKT cell-deficient mice strains (β_2_m^−/−^ and CD1d^−/−^) reported that NKT cells were dispensable for T cell-dependent allergic airway inflammation (52), even though AHR was not analyzed.

A possible reason to explain the discrepancies between studies concerning the implication of iNKT cells in asthma severity is that, in addition to iNKT cells, type II NKT cells were also absent in β_2_m^−/−^ and CD1d^−/−^ mice (53, 54), while β_2_m^−/−^ mice also lack CD8 T cells. Then, it is not excluded that the absence of type II NKT and CD8^+^ T cells could influence the effect of iNKT cells in asthma severity. Another point is that asthma symptoms are more severe in 129/Sv mice compared to BALB/c and C57BL/6 animals (55). The iNKT cell-deficient mice cited here (β_2_m^−/−^, CD1d^−/−^, and Jα18^−/−^ mice) were created on a 129/Sv background. Some results showing no significant differences in airway eosinophilia used 129/Sv × C57BL/6 CD1d^−/−^ mice (47), while those describing CD1d^−/−^ and Jα18^−/−^ mice as more resistant to asthma used CD1d^−/−^ and Jα18^−/−^ backcrossed with BALB/c animals (51). In our hands, Jα18^−/−^ (backcrossed at least 10 times in C57BL/6) presented lower allergen-induced airway inflammation and AHR than controls (50). Recently, Kronenberg’s team created a new mouse strain deficient for iNKT cells. These mice presented no airway eosinophilia and significantly less pulmonary resistance in response to OVA challenge than did their wild-type (WT) counterparts (56). Hence, the discrepancies reported may also result from a possible low number of backcross of the knockout mice used. Finally, the microbiota differences between the animal houses where the studies were performed cannot be excluded. In this context, an elegant study by Blumberg’s team showed that iNKT cells accumulated in the lung and in the colonic lamina propria in germ-free (GF) mice, rendering these animals more susceptible to OVA-induced asthma and oxazolone-induced colitis (57). The colonization of neonatal GF mice with a normal flora or *Bacteroides fragilis* decreased the number of iNKT cells and protected the mice against these diseases, clearly establishing a link between iNKT cells, the microbiota, and disease (57, 58).

These studies were highly informative but were designed to analyze a specific allergic asthma model. They, therefore, underestimated the complexity of asthma pathogenesis. It was subsequently shown that α-GalCer, the cognate antigen for iNKT cells, protects sensitized mice against asthma symptoms when administered 1 h before the first challenge (59). The mechanisms involved are dependent on IFNγ production by α-GalCer-stimulated iNKT cells (59). In another context, α-GalCer, administered i.n. at the time of sensitization, was found to act as an adjuvant, enhancing asthma symptoms (42). This study echoed those in non-human primates showing that the administration of α-GalCer alone induces AHR in monkeys (60). The iNKT cells are resident mostly in the intravascular space rather than in the pulmonary tissue itself, and they are rapidly mobilized after exposure to airborne lipid antigen, to which they respond by the secretion of cytokines (42). Thus, different lipid antigens in the airways, unrecognized by conventional T cells, may amplify airway inflammation by acting on iNKT cells.

Other asthma models have recently been used to investigate the role of iNKT cells. Intranasal administration of the natural House Dust Mite allergen without adjuvant has been shown to induce iNKT cell recruitment in the lung. The iNKT cells were stimulated *via* OX40–OX40 ligand interactions to generate a pathogenic Th2 cytokine environment (61). In this model, iNKT-deficient mice displayed significantly lower levels of pulmonary inflammation than WT mice (61). iNKT cells were further implicated in the model of asthma induced by *Aspergillus fumigatus* (62). This fungus, which is associated with a severe form of asthma, expresses asperamide-B, a glycolipid specifically recognized by both human and mouse iNKT cells (62). The i.n. administration of *A. fumigatus*- or asperamide-B rapidly induces AHR, by activating pulmonary iNKT cells in an IL-33-ST2- and IL-4/IL-13-dependent manner (62).

Overall, these findings indicate that iNKT cells promote allergic asthma inflammation and AHR principally through the secretion of IL-4 and IL-13. The Th2 paradigm explains many features of asthma, but this disease is not limited to pro-Th2 allergic immune responses and may also include a number of different phenotypes, such as neutrophilic asthma (63–65). In this context, the i.n. administration of α-GalCer activates IL-17-secreting iNKT (iNKT17) cells, which, in turn, recruit neutrophils to the airways (25). iNKT17 cells are also required for the pathogenic mechanism responsible for disease severity in the model of asthma induced by ozone, a major air pollutant (66). These findings indicate that iNKT2 and iNKT17 cell populations may contribute to asthma inflammation in different ways.

### iNKT Cells and Asthmatic Patients

Several studies have analyzed the possible implication of iNKT cells in the physiopathology of human asthma. Studies analyzing the frequency of iNKT cells in the [bronchoalveolar-lavage fluid (BALF)] or bronchial tissues of asthmatic patients have reported discordant results (67–70). The study of Akbari et al. (68) found that about 60% of the pulmonary CD4^+^CD3^+^ T cells in adult patients with moderate-to-severe persistent asthma were iNKT cells. These results were not reproduced by Vijayanand et al. (69), who found that up to 2% of the T cells obtained from airway biopsy, BALF, and sputum induction from subjects with mild or moderately severe asthma were iNKT cells. The study of Thomas et al. (71) also observed less than 2% of iNKT cells among gated T lymphocytes from BALF of asthmatic patients. Of note, further analysis from the initial group have demonstrated that only a small fraction of T cells in the lung of adult asthmatic patients were iNKT cells (72). In our hands (73), iNKT cells accounted for less than 1% of T cells in BALF from severe asthmatic children. The discrepancies with the first study (68) could be due to the limited number of samples, the heterogeneity of the cohort, or to non-specific staining of cells in BALF, as suggested by the study of Thomas et al. (71).

It was showed that the frequency of iNKT cells in the blood of adult asthmatic patients was similar to that in blood from control donors (74). Further, it was suggested that pro-Th2 iNKT cells may be particularly frequent in blood from asthmatic patients, and that these cells was associated with lung function (67). Our previous study indicated that the percentage of peripheral blood iNKT cells did not differ significantly between asthmatic children classified as exacerbators (1 or more severe exacerbations in the last 12 months) and those classified as non-exacerbators (75). Similarly, it has recently been reported that there is no relationship between the frequency of iNKT cells and that of IL-4- or IFNγ-producing iNKT cells in the blood of 1-year-old children and asthma-related clinical outcomes at the age of 7 years (76).

There is now a consensus that a limited number of iNKT cells is present in the BALF of adults and pediatric patients with severe asthma. However, several questions remain unanswered: Is the presence of iNKT cells in the BALF associated with specific asthma endotypes? What role do iNKT cells play in the pathophysiology of asthma? Further studies are therefore required to characterize the mechanisms by which iNKT cells could contribute to asthma.

## MAIT Cells

Invariant natural killer T and MAIT cells may be considered to be “twins” in several respects. Like iNKT cells, most MAIT cells express an invariant TCRα chain (Vα7.2-Jα33 or TRAV1-2-TRAJ33) and a small number of TCRβ chains (77). MAIT cells are restricted by the MHC class I-related molecule MR1 and recognize microbial-derived vitamin B2 (riboflavin) metabolites, such as the 5-(2-oxopropylideneamino)-6-d-ribitylaminouracil (5-OP-RU) (78). The endogenous ligands able to either select MAIT cells in the thymus or to potentially stimulate these cells in peripheral lymphoid organs remain to be defined.

Mucosal-associated invariant T cells, similar to iNKT cells, develop in the thymus, where they became functionally competent and able to produce IFNγ, TNFα, and IL-17 in response to stimulation (79). MAIT cells produce low-to-moderate levels of IL-4 and IL-13 when stimulated (80–83). Thymic MAIT development is also directed by the transcription factor PLZF and is dependent on microRNAs (79, 84). Human MAIT cells are CD8^+^, CD4^+^, or double-negative (CD4^−^CD8^−^) and express high levels of CD161 and IL-18Rα (85). MAIT cells can be activated in a TCR-dependent and -independent manner. In the latter situation, they can be stimulated by pro-inflammatory cytokines, such as IL-7, IL-12, IL-18, and IL-23 (29, 86, 87). Indeed, MAIT cells, in addition to IL-18Rα, can also express IL-7Rα, IL-23R, IL-12Rβ1, CCR5, CXCR6, CCR6 (83, 84, 88, 89). These receptors will allow the activation of MAIT cells by IL-7, IL-12, IL-18, and IL-23 and their migration to peripheral tissues.

Despite their striking similarities, iNKT and MAIT cells also differ in several important ways. Unlike iNKT cells, MAIT cells are rare in conventional laboratory mouse strains and abundant in humans. In healthy individuals, MAIT cells account for up to 10% of peripheral blood T cells and are numerous in the gut, lung, and liver (49, 85, 89). The expansion of the MAIT cell population in response to commensal flora antigens explains their abundance in mucosal tissues, in which they are involved in antimicrobial responses (49). Their presence in the liver may be explained by the constant exposure of this organ to bacterial products absorbed from the gut. There is a clear causal relationship between the number of MAIT cells and the presence and the diversity of the commensal flora, as shown by the absence of MAIT cells from the peripheral organs of GF mice (49).

### MAIT Cells and Infections

In addition to the commensal flora, pathogens may also stimulate MAIT cells, which play a crucial role in antimicrobial defenses, through the secretion of IFNγ, TNFα, and IL-17 and the killing of target cells through the production of cytotoxic perforin and granzyme B molecules (29, 87, 90). MAIT cell analysis, in both humans and in experimental models, has been greatly facilitated by the use of antigen-loaded MR1 tetramers (83, 84). Experimental studies in non-human primates have reported the activation of circulating MAIT cells in response to Bacillus Calmette–Guerin vaccination and *Mycobacterium tuberculosis* infection (91). MAIT cells from the spleen of these macaques produced IFNγ, TNFα in response to stimulation by *Escherichia coli* in a TCR-dependent manner (91). Intranasal inoculation with *Salmonella typhimurium* in mice induced a striking enrichment in IL-17-producing MAIT cells in the lungs (92). The response of MAIT cells to lung infection with *S. typhimurium* was rapid and dependent on the MR1 presentation of riboflavin biosynthesis-derived bacterial ligands (92). These findings are consistent with previous reports indicating that patients infected with mycobacteria have many more MAIT cells in the infected lung and fewer MAIT cells in the blood than uninfected controls (93, 94).

Infections with viruses, such as dengue virus, hepatitis C virus, influenza A virus, and HIV-1 can activate human MAIT cells. MAIT cells do not recognize virus antigens, because no riboflavin metabolites are found in host cells or viruses (78), but they may be activated by cytokines produced during viral infection, such as IL-18 in synergy with IL-12, IL-15, and/or IFNα/β (29, 95). Activated MAIT cells during virus infections robustly secrete IFNγ and granzyme B (29, 95).

Mucosal-associated invariant T cells have also been implicated in non-infectious diseases. Several studies have reported large decreases in MAIT cell number in the peripheral blood of patients with the following diseases: antineutrophil cytoplasm antibody-associated vasculitis, chronic kidney disease, Crohn’s disease, ulcerative colitis, newly diagnosed and relapsed multiple myeloma, obesity and type 2 diabetes (96–100). However, the mechanisms by which MAIT cells influence these human diseases remain to be elucidated.

### MAIT Cells and Adult Asthmatic Patients

Despite the prevalence of MAIT cells in the lung, and their involvement in airway infections, very little is known about the possible role of these cells in asthma. MAIT cells are detected in human fetal lung and are numerous in the lungs of adult rhesus macaques (91, 101), consistent with a protective role against infections in this organ. The frequency of MAIT cells is significantly lower in the peripheral blood, sputum, and bronchial biopsy specimens of asthmatic patients than in control subjects (102). The percentage of MAIT cells in BALF does not differ significantly between these two groups (102). A re-analysis of the results, comparing patients with mild, moderate, or severe asthma to healthy donors, showed that the lower frequency of MAIT cells was significant only in the peripheral blood and sputum of patients with moderate or severe asthma (102). The results of this study suggest that the frequency of MAIT cells is negatively correlated with clinical severity. Furthermore, MAIT cell frequency is associated with serum vitamin D3 concentrations and the use of oral corticosteroids. Proof of concept for the association between corticosteroid use and MAIT frequencies was provided by the demonstration of a decrease in the frequency of circulating MAIT cells in 12 patients with moderate asthma treated with oral corticosteroids for seven days (102). It remains to elucidate whether corticosteroids may modify MR1 expression and MAIT cell activation. Some drugs can influence antigen presentation by MR1 molecules. For instance, doxofylline, a bronchodilator used to treat asthma, is known to upregulate MR1 expression weakly, but does not act as a MAIT cell agonist (103). Thus, some of the drugs currently used in asthma treatment may influence MAIT cell functions.

Mucosal-associated invariant T cells are present and can be activated in the lung (Figure 1). However, to date, there is no evidence indicating that MAIT cells could recognize *via* their TCR any airway allergens or pollutants potentially implicated on asthma. Consequently, MAIT cells could be activated either directly by endogenous compounds presented by MR1 molecules or indirectly by pro-inflammatory cytokines present in the lung of asthmatics, namely IL-1β, IL-7, and IL-23 (104, 105). These two possibilities are not mutually exclusive. Of note, asthma exacerbations are frequently associated with virus infections (104), which indirectly activate MAIT cells through the induction of pro-inflammatory cytokines (29, 95). MAIT cells then activated will secrete IFNγ and/or IL-17. Knowing that MAIT cells secrete low levels of Th2 cytokines, namely IL-4 and IL-13 (80–82, 88–90, 106), we cannot exclude the possibility that these lymphocytes will promote Th2 responses (Figure 1). However, as discussed before, asthma is a complex pathology that is not restricted to overproduction of Th2 cytokines. Further, IL-17 and IFNγ production were associated with asthma severity in some steroid-resistant patients (107, 108). Overall, these studies have provided a basis for further analyses of the role of MAIT cells in asthma, potentially on steroid-resistant asthma, and of the mechanisms by which these cells affect asthma severity.

### MAIT Cells and Pediatric Asthmatic Patients

Asthma is frequent in children, but little is known about the possible influence of MAIT cells on the pathophysiology of this disease in childhood. We recently reported a similar frequency of circulating MAIT cells between exacerbators and non-exacerbators, in a population of asthmatic children (75). However, the frequency of IL-17-producing MAIT (MAIT-17) cells was found to be positively correlated with the number of severe exacerbations and negatively correlated with the asthma control test (ACT) score (75). No significant modification of the frequency of IFNγ-producing MAIT cells was observed (109). These findings indicate a possible association of MAIT-17 cells with asthma symptoms. Interestingly, higher levels of IL-17 production by MAIT cells have been observed in a number of non-infections pathologies, such as obesity, type 2 diabetes, and inflammatory bowel disease (99, 100), indicating that mechanisms other than infections may favor IL-17 production by MAIT cells.

Another recent study reported that an association between a high frequency of circulating MAIT cells at 1 year of age and a lower risk of asthma by the age of 7 years (76). Furthermore, this high frequency of MAIT cells was correlated with higher frequency of IFN-γ-producing CD4^+^ T cells, indicating a possible protective effect of MAIT cells as children grow older (76). IL-17 production by MAIT cells did not correlate with asthma in this study (110). Taken together, the results of these two studies suggest that MAIT-17 cells may be associated with asthma symptoms, whereas pro-Th1 MAIT cells may promote protection (75, 76).

## Conclusion

Our understanding of the biology of both iNKT and MAIT cells and their role in asthma has increased considerably in recent years (Figure 1). As a result, many new questions have been raised concerning the mechanisms by which iNKT and MAIT cells could promote human severe asthma. For example, time may be an important element, because asthma often begins early in childhood, when the number and functional properties of lung iNKT and MAIT cells may be fixed. Studies conducted in children may, therefore, be crucial. Analyses of circulating iNKT and MAIT cells, as biomarkers, may be informative, but data for BALF and bronchial biopsies are still lacking. Finally, detailed analyses of the frequency and functional subsets of these cells in the context of different asthma endotypes may be crucial for the development of new therapeutic approaches.

## Author Contributions

GL and MLM wrote the manuscript.

## Conflict of Interest Statement

The authors declare that the research was conducted in the absence of any commercial or financial relationships that could be construed as a potential conflict of interest.
